# Circular Policy: A New Approach to Vector and Vector-Borne Diseases’ Management in Line with the Global Vector Control Response (2017–2030)

**DOI:** 10.3390/tropicalmed7070125

**Published:** 2022-07-04

**Authors:** Christiana Tourapi, Constantinos Tsioutis

**Affiliations:** 1 Departments of Health Sciences and Medicine, European University Cyprus, Nicosia 2404, Cyprus; ct212291@students.euc.ac.cy; 2 School of Medicine, European University Cyprus, Nicosia 2404, Cyprus

**Keywords:** vector, vector-borne disease, Integrated Vector Management, GVCR, climate change, circular policy

## Abstract

Integrated Vector Management (IVM) has yielded exemplary results in combating and preventing vector-borne diseases (VBDs) and their vectors. It’s success and positive outcomes depend on the sound planning, implementation, enforcement, and validation of the locally adapted vector control efforts from the involved national sectors and stakeholders. Nevertheless, current realities create several implications impeding IVM’s performance. Hence, there is a need to adjust local IVM plans to several factors, such as (i) the rapidly changing and unpredictable environmental conditions (i.e., climate change, shift on species distribution, invasive species—*Anopheles stephensi*, *Aedes aegypti* and *Ae. albopictus*); (ii) the environmental impacts from human activities (i.e., fossil fuel use, food sources, industry, land use, urbanization and deforestation); (iii) changes in human demographics and the international movement of people (travelers and forcibly displaced persons due to conflicts and severe weather) increasing the risk of contracting and transmitting vector-borne diseases and shifting humanitarian emergencies and societal demands; (iv) the SARS-CoV2 pandemic outbreak and the implication on national public health systems; (v) the continuous flow of technological advancements and newly acquired knowledge; (vi) the realization of the strong link between planetary health and public health. Addressing these factors in IVM can become difficult, taking into consideration the numerous involved sectors, stakeholders, and fields in the management of vectors and vector-borne diseases (VBD). This document proposes and discusses the aspects and steps of a holistic approach, referenced as the Circular Policy, for national and local IVM strategies to be effective and adaptable, capable of providing the optimum outcomes.

## 1. Introduction

The World Health Organization (WHO) has developed a strategic approach, the Global Vector Control Response 2017–2030 (GVCR), to fight both vector and vector-borne diseases (VBDs), as it estimates that near 80% of the human population is at risk to contract at least one VBD in their lifetime, and more than 700,000 people lose their lives annually [1]. GVCR’s targets are that, by the year 2030: mortality due to VBDs is to be reduced by at least 75% compared to 2016; the case incidence due to VBDs should be reduced by at least 60% compared to 2016; epidemics of VBDs should be prevented in all countries [1]. To reduce the threat of VBDs affecting humans and ease the burden of public health systems, the GVCR Framework sets as a foundation the enhancement of national vector control capacity and capability and the improvement of basic and applied research and innovation. Built on these foundations are four pillars of action: (a) strengthening of intersectoral and intrasectoral actions and collaborations, (b) public engagement and community mobilization, (c) enhancement of monitoring and surveillance efforts of vectors combined with the evaluation of the interventions and (d) scaling up vector control approaches and integrating tools in their management [1,2].

In 2017, the WHO published the ‘Framework for a National Vector Control Needs Assessment’ to enable its Member Countries to evaluate their current situation on vector and VBDs control and redirect their national policies and strategies based on identified gaps and/or opportunities and to reach the GVCR’s targets [2]. It aims to guide planning and resource utilization for nations to implement Vector Control Strategic Plans in line with the GVCR [1,2].

GVCR Framework’s urgency arises from ‘Social, demographic and environmental factors have altered pathogen transmission patterns, resulting in intensification, geographical spread, re-emergence or extension of transmission seasons (…) The dynamic and complex nature of vector-borne pathogens complicates predictions of the impact of existing, re-emerging or new diseases on human health (…) This complexity and unpredictability underscores the critical need for adaptive and sustained approaches to prevent and reduce pathogen transmission to reduce disease burden’ [1].

Additionally, the WHO recognizes IVM’s—‘a rational decision-making process for the optimal use of resources for vector control’—significant contribution to the prevention and control of VBDs, due to its cost–effectiveness, intersectoral actions, incorporation of regulatory and operational measures in vector control, the involvement of local communities, the locally applied decision-making on vector control actions, and its alignment with sustainability goals [3].

Factors that can impact vectors’ ecology such as their geographical and seasonal distribution) and their transmission dynamics (i.e., increase of infection risks from disease-causing pathogens) are deforestation, urbanization, changing land use patterns, water control projects, loss of biodiversity, introduction of alien species, agriculture, and increased global travel, human migration and trade, and insecticide and larvicide resistance in all insect groups that serve as vectors of emerging diseases [4,5,6,7,8,9,10,11,12,13,14,15].

The rapidly changing VBDs scenery affects human sustainability from a socioeconomical point of view by burdening healthcare systems, by creating social and health inequities, by impacting vital economic sectors such as tourism and labor, and by reducing socioeconomic development, especially in areas with the poorest populations (i.e., malaria is often considered a barrier to economic development) [1,2,16,17,18,19].

When developing policies for the prevention and control of vector and/or VBDs, national authorities are advised to base them on four fundamental pillars which are: the surveillance and monitoring of vector populations and their larval habitats, pathogen screening processes of vectors and/or sentinel animals or saliva capture techniques, interventions for prevention and/or control, and the evaluation of the aforementioned efforts [20,21,22,23]. The rapid and unpredictable shift in the VBDs’ transmission and the dispersal of vectors carrying and transmitting pathogens obligates nations to constantly evaluate their implemented measures. Not reevaluating and realigning the original policies with current needs and not assessing the success and outcomes of the interventions may prove detrimental to VBD transmission and outbreaks. Hence, the Circular Policy approach is suggested to be applied in order for nations to achieve the GVCR’s optimistic targets by incorporating evaluation and auditing after each full cycle of preventative and/or control actions.

The current review proposes and describes the workflow of the Circular Policy approach—a tool in response to the rapidly changing dynamics of vectors and VBDs that is based on ‘Planning, Acting, Evaluating and Reacting’. National authorities can benefit from this approach, enabling them to adjust and align existing drafted policies and action plans to current needs, trends, and opportunities, to minimize the risk of emerging or re-emerging diseases and epidemics.

The Circular Policy approach cannot stand alone without the guiding frameworks, and managerial tools (Table 1), already developed by organizations with decades of experience, such us the WHO, the European Centre for Disease Prevention and Control (ECDC), the Centers for Disease Control and Prevention (CDC), the United Nations Development Programme (UNDP), the International Atomic Energy Agency (IAEA), and the Food and Agriculture Organization of the United Nations (FAO). The legislative framework surrounding the rational and legal use of chemicals to combat vectors is the same for all EU Member countries (with noticeable different accession rates into the national legislations) but differs among other countries, and this should be examined and taken into consideration during IVM planning.

## 2. Discussion

IVM promotes the effective collaboration between the health sector and other involved public and private sectors, as well as the engagement and mobilization of communities. As described in the Handbook for Integrated Vector Management (2012) [20] and the Global Strategic Framework for Integrated Vector Management (2004) [23], published by the WHO, key elements of an IVM strategy are (i) advocacy, social mobilization, and legislation; (ii) collaboration within the health sector and with other sectors; (iii) an integrated approach ensuring the rational use of available resources; (iv) evidence-based decision making; (v) capacity and capability building [20,23].

The first step in applying the Circular Policy approach for the effective implementation of IVM is to recognize the involved sectors, stakeholders, and areas of expertise and their contribution in vector and VBD control.

(A) Identified Sectors and fields of expertise involved in Vector and/or VBDs prevention, control, and treatment

The identified sectors and fields of expertise responsible for planning, creating, establishing, implementing, enforcing, auditing, and disseminating an effective and sustainable IVM strategy as outlined in the Framework for a National Vector Control Needs Assessment (2017) [2] and the GVCR Framework (2017) [1] are:

1. Political commitment from parties: can be the driving force that will embark the management cycle of an integrated management plan. Endorsement and support by governing authorities is a necessary step towards the implementation of a successful IVM strategy [2].
2. International and national policies and legislations: this is pivotal to the creation of sound strategies and their subsequent enforcement. Sub-national levels and local governments must follow guidance documents (policies created by governmental bodies that interpret laws and regulations) to implement the national IVM strategy. In some countries the private sector is recruited in the battle of vector and VBD control and can often follow organizational policies (formal policies adopted by businesses, organizations, and non-governmental entities) that address how they operate, and how may impact their employees, members, volunteers etc. [53,54]. International regulations are succeeded into national laws of the signee parties, e.g., International Health Regulations [55], European Directives and regulations apply for all EU Member States, and Communicable Diseases Laws for each nation are applied during vector and VBD control activities.
3. Institution enforcement—Managerial aspect [56]: the key elements of the IVM strategy are managed; competent authorities are assigned with clear goals and targets to manage the prevention, control and treatment of VBDs.
4. Institution enforcement—Technical facilities/infrastructure/staff [2]: the managerial aspect of the IVM cannot function without the strengthening of technical facilities, infrastructure, and staff, and is an inclusive process.
5. Intrasectoral and intersectoral collaboration [2]: since each sector and field of expertise in IVM depend on the efforts and results that each has, it is crucial to have a transparency of actions, reporting, and intersectoral meetings to avoid the overlapping of efforts or gaps in implemented actions. This is achieved with the formation of coordinating committees with regular meetings and the exchange of information on recognized opportunities and challenges, in order to achieve Best Practice Management. In turn, meetings with subcommittee representatives and relevant governmental committees should take place, to inform and report on the progress. Reviewing and evaluating of all activities can be performed annually and action plans may be amended if needed based on the assessed needs and gaps. After the primary evaluation and reporting, the assigned overarching authority retrieves financial resources for the activities and allocates the required funds to each competent sector/department/division. Since vectors and VBDs know no borders, collaborations with other nations when shared interests and resources are at risk, these can be safeguarded through international agreements.
6. Data Sharing [2]: this section cannot be enforced without the prior strengthening of institution management and infrastructure and without intersectoral cooperation. It concerns the collection of data (e.g., surveillance and monitoring data); recording of data; application of data systems; reporting of data to higher level officers and/or committees and sharing data with all involved stakeholders and the public.
7. Evaluation of efforts: evaluation of a national plan or program and political commitment/policy are among the most important pillars for integrated implementation. What is a plan good for, when it is not performing as it should or is not adaptable to seasonal, social economic and/or capacity needs of each involved level? An evaluation framework and the selection of evaluators (evaluating authorities) must be set prior to the plan’s implementation and be specific to the plan’s intended efforts (specific metrics/indicators) to achieve the desired result. This action is often overlooked or not set from the beginning, resulting in the blindfolded intensification of interventions (physical, chemical, biological) when the desired outcomes are not achieved.
  - Why is it necessary: to ensure the fulfillment of the key principles of IVM and guide public health activities i.e., decision-making processes based on scientific data analysis, social equity from the specific actions/measures, effective performance of the involved sectors, and establishing efforts based on the desired result and accountability [57].
  - How is this achieved [58,59]: by setting feedback systems and practical evaluations, ensuring learning and the further improvement of the strategic plan. Evaluations are conducted routinely to provide valid and detailed information to the Inter-Ministerial Steering Committee (overarching authority) to manage and effectively implement the national vector control program. For an evaluation framework to be developed, contribution is required from evaluation experts, directors and staff, governmental officials, independent control associates, researchers, and institutions involved in vector research. Furthermore, impact assessment surveys for vector control programs must include evaluation systems to identify and analyze potential adverse effects (environmental impacts on ecosystems and /or other beneficial species), which may require policy adjustment in order to be avoided in the future and/or mitigated.

The evaluators must be trusted persons who are credible and knowledgeable with evaluating processes or systems. They must be impartial when performing intervention evaluations and must follow up on all internal personnel evaluations for transparency purposes. Collaborative evaluation approaches are characterized by increased responsiveness and commitment, enhanced motivation of achieving objectives, the delivery of a wide range of evaluation skills, and can further create and establish partnerships [58,60].

8. Overarching authority: an overarching authority (i.e., ISC) coordinates actions, allocates responsibilities, overviews activities, and evaluates the overall results. It is often recommended that all governmental bodies having jurisdiction in this field are included in the established overarching authority. However, the overarching authority members are not exclusively limited to higher administration levels. Representatives from all relative management working groups are important to participate in the authority, as they are competent to provide advice, data, scientific information, and insight on the program’s processes and outcomes [2].
9. Communication, awareness, mobilization, education, and engagement [61]: this field is for planning the communication of a new or amended national policy and/or strategy and is performed at two different levels: the sectorial level, between the ISC and the subnational, academic, institutional level, and the private sector, and to the public, which is the general population. For a successful communication strategy, setting objectives, planning, and developing logical and rational interventions and measurements (indicators) of said communication activity—either being complex (i.e., campaigns, workshops, training) or single (i.e., conferences, newsletters, websites, public relations events, press events, social media, smartphone applications, publications etc.) interventions—is crucial [61].
10. Financial resources: sufficient funding is necessary to achieve the above. Managerial efforts involve expertized personnel, specialized equipment, sound infrastructure, and procured services. Dedicated funding to all sectors with appropriate resources can secure the outcome of an IVM strategy [2].

(B) Circular Policy Approach—Suggested Workflow to Achieve Optimum Implementation Results of an IVM

The Circular Policy approach was conceptualized upon the drafting of The Republic of Cyprus’ (RoC) Situation Analysis and Vector Control Needs Assessment (VCNA) reports for WHO, in 2021.

A meticulous study of RoC’s current situation on vector and VBDs control was performed and all available data from governmental departments, research institutions and other involved stakeholders were gathered and evaluated. Both reports followed the recommendations and the instructions outlined in the Framework for a National Vector Control Needs Assessment (2017, Annex I) [2]: ‘This Framework has therefore been developed to assist programmes in adopting the GVCR guidance for more effective and sustainable vector control. The Framework provides a practical tool to help programmes better understand their situation and document their needs both for baseline assessment and for progress tracking. This assessment process will enable Member States to generate or revise their Vector Control Strategic Plan in line with the GVCR and mobilize domestic or external resources to address identified needs’ [2].

The RoC was the first country to submit the Situation Analysis and VCNA reports, followed by Croatia, and were both presented in the ‘Regional Training Course on National Vector Control Needs Assessment in Preparation of Integrated Vector Management including the Sterile Insect Technique’ (Limassol, Cyprus, 9–13 May 2022). The training course was organized by the International Atomic Energy Agency (IAEA) within the framework of the IAEA Regional Technical Co-operation Project RER/5/026: ‘Enhancing the Capacity to Integrate Sterile Insect Technique in the Effective Management of Invasive Aedes Mosquitoes’.

**Figure 1:**
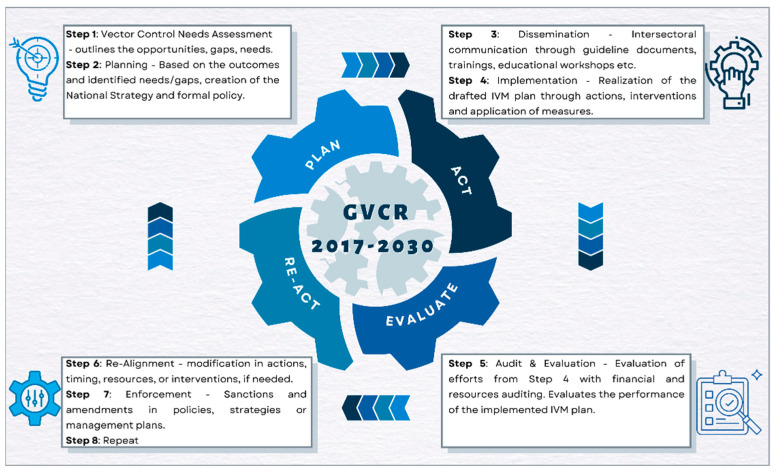
The Circular Policy approach.

The Circular Policy approach is constituted by four categories, Planning–Acting–Evaluating–Reacting, with eight steps in total, as shown in Figure 1.

(A) Plan

Step 1: Assessment of the current situation

International organizations such as the WHO, the IAEA, and the United Nations Development Program (UNDP) often urge Member Countries (MC) to perform assessments of current national and subnational needs prior to the implementation of strategies. The assessment is always performed by independent parties (assessors), delivering reports concerning the situation analysis and the identification of opportunities and gaps of the matter of interest. By identifying the opportunities created by already-established measures and the gaps/needs that will help MC to comply with the drafted strategies, more targeted national policies can be created to facilitate their implementation. The situation analysis and needs assessment reports are delivered to the highest rank committee for reviewing.

Step 2: Planning

After receiving the Situation Analysis and Needs Assessment Reports, the overarching authority is responsible for the following: conducting approvements of suggested tools; appraising submitted reports and coming into agreement for the policy and strategies needed for implementation; drawing a proposed National Strategic Plan or national policy, which can be drafted in order to accommodate each nation’s needs on IVM; supervising and directing the adoption of Strategic Plans and their implementation processes; safeguard their outcomes by making appropriate adjustments.

To minimize miscommunication, lack of cooperation, lack of clear responsibilities and duties, and ensure funds’ allocation to each sector, the Circular Policy proposes the creation of subcommittees, as follows:

1. Vector Research Sub-committee, responsible for coordinating activities regarding vector research, surveillance, and monitoring. An annual report on the results and required funds can be created submitted to the competent authority for approval.
2. VBD and Public Health Sub-committee can be responsible for the coordinated activities regarding VBDs and public health issues related to vector communicable diseases.
3. VBD Wildlife—Environmental Health Sub-committee. VBDs can affect animals as much as humans and both enzootic and zoonotic diseases may later pose a threat for human health as well. It can be responsible for coordinating research studies and the surveillance and monitoring of wildlife populations that are at risk of VBDs, as well as domesticated animals (for *Dirofillaria* spp., leishmaniasis etc.) and livestock animals (equine encephalitis etc.).
4. Biocides’ Regulations Coordinating Sub-committee to oversee all relevant procedures of the regulating, authorizing, and licensing biocides/control—the storage and transport, distribution, and repackaging of chemicals/regulative, importing, exporting, circulating, storing, and repackaging activities of vector control biocides substances for public health.
5. Implementation Sub-committee, where all vector control efforts (measures and interventions) should be managed. After consulting with the Vector Research Sub-committee, semester programs can be drafted allocating vector control activities and areas to be treated with physical, biological, or chemical means. The application of biocides can be conducted based on these programs for all vector species targeted and specific, minimizing fruitless actions and environmental impacts.
6. Public Engagement Sub-Committee, where communication specialists are suggested to be involved, especially when communicating with the public, to coordinate all involved sectors in preparing publications, press releases, and dissemination of informative material, which is essential for public engagement. Furthermore, it is suggested to be responsible for the creation and keeping of a Scientific Registry of vector and entomological experts.

In turn, all the above-mentioned sub-committees will have an open line of communication with the overarching authority and their results (budget, reports, statistics, data, trends etc.) can be advised for decision-making.

(B) Acting

Step 3: Dissemination

Planning needs to be performed for all involved and affected sectors/stakeholders, hence policies, strategies, action plans, and relative frameworks and legislations are required to be disseminated among them. Guideline documents, trainings, educational workshops, and certifications can be performed to achieve the maximum knowledge transfer to all operational levels.

Step 4: Implementation

“Use and strengthen what there is already available and create what is missing” policy. Based on the Situation Analysis and VCNA sectors that need strengthening, it is suggested that they be provided with the appropriate funds and resources to be upgraded. Then, they will be able to perform their responsibilities and obligations that have already been established in a clear and comprehensible manner. Each operational sector (public or private) begins with the implementation of the drafted managerial and/or control plans. Recording of the efforts is performed at this step, which at a later stage will be collated into reports by the designated sub-committee to be reviewed by the evaluation and auditing teams and the overarching authority.

(C) Evaluating

Step 5: Audit—Evaluation

One of the most important and integral parts of the Circular Policy is the evaluation of efforts and auditing of the financial aspects of every IVM. Many national IVM programs may run for years without any evaluation, resulting in a lack of knowledge of performance and effectiveness, and can even lead to misguided decisions. No IVM Program can run without the surveillance and monitoring of vectors and VBDs, which in turn will provide data-based evidence for evaluation during the progress of the program. Of course, evaluation systems must be compliant with the plan’s targets and objectives, must be useful and not create obstacles to vector control efforts, and above all fill gaps and needs in an already established program or plan. The best practice code is advised to ensure democratic accountability. Financial auditing is also crucial at this point, to evaluate funds’ allocation and resources used for the complete implementation of any suggested IVM Program.

Evaluation and auditing will provide insight regarding efforts and actions to prevent or combat vector blooming and VBD epidemics. The evaluation and auding teams need to draft detailed reports to be reviewed by the overarching authority, based on the evaluating criteria of the effectiveness, efficiency, sustainability, relevance and usefulness of all actions and funds. Furthermore, their scope can be of institutional, temporal, sectoral and of a geographical range [58,62]. Evaluation and auditing reports are suggested to be performed on an annual basis.

(D) Reacting

Step 6: Re-Alignment

If the sub-committees, the evaluation, and auditing teams suggest in their reports that there no further action or amendment is needed, then the overarching authority can consult this decision. If all evidence suggests that a modification in actions, timing, resources, or interventions is needed, the overarching authority may consult this conclusion and act accordingly.

Step 7: Enforcement

If any unlawful discrepancies or intentional misuse of resources and/or funds is documented, or failure to implement measures that may result to bridging public health safeguarding, then the national overarching authorities must report this to the international organizations that may have distributed funds and enforce sanctions to the offenders.

Step 8: Repeat

Any formal changes can then be planned, disseminated, and implemented by the lower operational levels and then be evaluated and audited anew in order to maximize IVM’s outcomes and efficiency to reach the set targets outlined in the GVCR 2017-2030 [1].

## 3. Conclusions

The implementation of the IVM will benefit from the application of the Circular Policy approach, as it allows decision makers to recognize the gaps and needs of a vector and VBD control program, resulting in the optimal allocation of time, resources, and financial assets. Evaluation plays a crucial role in Circular Policy as it has been shown that evaluation plans and feedback positively affect a plan’s effectiveness. By integrating evaluation systems to drafted policies and becoming a key component in decision-making processes, IVM plans can be readjusted to the current needs and factors that may affect its success. The evaluation of efforts can improve policies over time by aligning them with their cycle (formulation—planning—resource allocation—program design—implementation—delivery of results) steering them to the best possible direction. Furthermore, evaluation contributes to the designing stage of an IVM plan, since collated information from a wide range of involved stakeholders can provide more clear choices to the decision makers and guide them to prioritize identified needs and gaps. The selection of the most-suited instrument or measure is facilitated and provide support for ‘mid-course correction’ [57].

Every nation’s concern is to safeguard public health but also to ensure socioeconomic growth and development. Implementing and following all the available frameworks and guidelines regarding vector and VBDs’ management can become overwhelming and cause confusion among the involved sectors. Hence, when setting clear national aims and objectives, followed by rational targets adapted to each country’s needs and opportunities, IVM will become the key to promoting One Health. One Health is ‘the collaborative effort of multiple disciplines—working locally, nationally, and globally—to attain optimal health for people, animals and our environment’ [63].

The Circular Policy approach can guide and facilitate the overarching authorities to implement IVM strategies that are both effective and agile to changes.

## Figures and Tables

**Table 1:** Frameworks, guidelines, reviews, manuals, and protocols published by International Organizations and their application sectors.

Organization	Documents	Sector
WHO	Latest Meetings of the WHO Vector Control Advisory Group [24]	Product developers, innovators, and researchers on the generation of epidemiological data and study designs to enable assessment of the public health value of new vector control interventions
	Vector control interventions designed to control malaria in complex humanitarian emergencies and in response to natural disasters [25]	
	Guidance framework for testing the sterile insect technique as a vector control tool against aedes-borne diseases [26]	
	Global vector control response 2017–2030: A strategic approach to tackle vector-borne diseases [1]	National, subnational, regional, and departmental level (i.e., governments and public health agencies, vector control workers)
	Norms, standards and processes underpinning development of WHO recommendations on vector control [27]	
	Equipment for vector control—Third edition [28]	
	Manual on environmental management for mosquito control, with special emphasis on malaria vectors [29]	
	Equipment for vector control specification guidelines—Revised edition [30]	
	Global vector control response: progress in planning and implementation [31]	
	Protecting the health and safety of workers in emergency vector control of Aedes mosquitoes: Interim guidance for vector control and health workers [32]	
	Integrating vector control across diseases [33]	
	The evaluation process for vector control products [34]	
	Framework for a national vector control needs assessment [2]	
	Handbook for integrated vector management [20]	
	Global Strategic Framework for Integrated Vector Management World Health Organization [23]	
	Keeping the vector out: housing improvements for vector control and sustainable development [35]	Communities
ECDC	Organization of vector surveillance and control in Europe [36]	National, subnational, regional, and departmental level (i.e., governments and public health agencies, vector control workers)
	Vector control practices and strategies against West Nile virus [37]	
	Integrated surveillance for prevention and control of emerging vector-borne diseases in Europe [38]	
	The importance of vector abundance and seasonality [39]	
	A spatial modeling method for vector surveillance [40]	
	Organization of vector surveillance and control in Europe [36]	
	Guidelines for the surveillance of native mosquitoes in Europe [41]	
	Guidelines for the surveillance of invasive mosquitoes in Europe [42]	
	Guidelines for presentation of surveillance data [43]	
	Field sampling methods for mosquitoes, sandflies, biting midges and ticks [22]	
	Core competencies in applied infectious disease epidemiology in Europe [44]	For training needs assessments in public health institutions
IAEA	Insect–pest control: Manuals and protocols [45]	Product developers, innovators, and researchers on the generation of epidemiological data and study designs to enable assessment of the public health value of new vector control interventions
	Guidelines for Studies on Plant-Based Vector Control Agents. In Traditional Medicinal Plants and Malaria [46]	
	Guidance framework for testing the sterile insect technique as a vector control tool against aedes-borne diseases [26]	
	Alternative vector control methods to manage the Zika virus outbreak: more haste, less speed [47]	National, subnational, regional, and departmental level (i.e., governments and public health agencies, vector control workers)
FAO	Ticks and tick-borne diseases selected articles from the WORLD ANIMAL REVIEW [48]	For training needs assessments in public health institutions
	Recognizing Rift Valley Fever [49]	
	Tsetse and trypanosomiasis information: Quarterly [50]	
UNE, STIP, ECSA	Real-Time Targeted Vector Mosquito Monitoring Best Practices Guide [51]	Citizen science
	Vector Mosquito Monitoring Via Biodiversity Specimen/DNA Data Sharing Best Practices Guide [52]	
UNDP	Multisectoral action framework for malaria [19]	National, subnational, regional, and departmental level (i.e., governments and public health agencies, vector control workers)

## Data Availability

Not applicable.
